# Urban plums and toads: do fleshy fruits affect the post-metamorphic growth of amphibians?

**DOI:** 10.7717/peerj.6337

**Published:** 2019-01-30

**Authors:** Mikołaj Kaczmarski, Piotr Tryjanowski, Anna Maria Kubicka

**Affiliations:** Department of Zoology, Poznań University of Life Sciences, Poznań, Wielkopolska, Poland

**Keywords:** Anura, *Prunus cerasifera*, *Bufotes viridis*, Development dynamics, Fruit trees

## Abstract

**Background:**

The main aim of the study was to analyse the influence of fleshy fruits (plums) on the post-metamorphic growth and feeding behaviour of the green toad *Bufotes viridis*. We tested the following two hypotheses: (1) juveniles of the green toad are characterised by faster growth in conditions involving fallen plums *Prunus cerasifera* due to the associated presence of more varied food such as invertebrates; (2) green toads exhibit more active feeding behaviour in the presence of fleshy fruits.

**Methods:**

A total of 120 fresh metamorphs of the green toad were randomly assigned to one of four groups: two experimental groups with fleshy plums and two other groups as controls (without fruits). Each group was kept in an enclosure to which wild invertebrates had free access. Each individual toad was measured for snout-vent length (mm) and body mass (g) every other day for 30 days. In order to determine whether fallen plums influence the feeding behaviour of toads, the number of active and hidden (under an artificial shelter) individuals was also noted.

**Results:**

The results showed that green toads from both enclosures with plums were characterised by more rapid growth than individuals from the control treatments. Simultaneously, in the enclosure with fleshy fruits, greater species richness of wild invertebrates was observed. No differences in active feeding behaviour were noted between control groups and groups with plums.

**Discussion:**

Fleshy fruits, upon falling, attract many types of invertebrates; thus they may represent good dietary supplements for fresh amphibian metamorphs. Therefore, the presence of fruit trees close to a breeding site might influences the post-metamorphic growth of amphibians, but not their feeding behaviour. The presence of insects associated with fallen fruit seems to favour the occurrence of amphibian populations, which is particularly important, since, due to political and social pressure, numbers of fruit trees are currently being reduced. To our knowledge, no previous study has investigated the potential influence of the presence of fruit trees on the growth and behaviour of anurans.

## Introduction

Usually, fruit trees lie outside the area of interest of ecologists, however, on the landscape level, the number of fruit trees is decreasing steadily (Orłowski & Nowak, 2007; Plieninger et al., 2012). Very few studies in this area show that the distribution of the greatest numbers of fleshy-fruited species is closely related to man-made components of the environment such as roads (Suhonen et al., 2017), orchards, rural areas (Suhonen & Jokimäki, 2015), and even electricity pylons (Dylewski et al., 2017).

Nonetheless fleshy fruits, as an attractive source of nutrients, are consumed by humans (Clark & Nicholas, 2013), invertebrates (Sallabanks & Courtney, 1992; Bieber, Silva & Oliveira, 2013; Rodríguez, Alquézar & Peña, 2013) and by many vertebrates such as lizards (Pietrzak, Steindorff de Arruda & Cechin, 2014), mammals (Virgós et al., 2010; Kurek, 2015; Lim et al., 2017), and, especially, birds (Jordano, 1994; Lázaro, Mark & Olesen, 2005; Jordaan, Johnson & Downs, 2011; Suhonen & Jokimäki, 2015; Suhonen et al., 2017). However, while birds and bats may eat ripe fruit directly from trees (Suhonen et al., 2017), fruit becomes an available resource for the majority of mammals or lizards only after it falls. Fruits lying on the ground decompose and attract numerous invertebrates by odour (Sallabanks & Courtney, 1992; Rodríguez, Alquézar & Peña, 2013) and colour (Fadzly & Burns, 2010). Moreover, some indirect interactions have also been described whereby predators (birds or mammals) deliberately choose fruits infested by insects. These tritrophic interactions are often unpredictable and difficult to measure (Rodríguez, Alquézar & Peña, 2013); however, fallen or rotten fruits lying on the ground may have implications for the functioning of the ecosystem.

Fallen fruits near amphibian breeding sites (e.g. different kinds of water bodies) may have important ecological effects and may constitute an undervalued component of the environment. This is because fruits attract insects (Sallabanks & Courtney, 1992) that can be eaten by amphibians. Following metamorphosis, which is a critical stage of life, amphibians begin intensive feeding prior to their first hibernation (Hartel et al., 2010; Schmidt, Hodl & Schaub, 2012). Physical growth during the first month of terrestrial life, as well as, in effect, post-metamorphosis size, affect life performance in the subsequent stage (Werner, 1986). Furthermore, body length and body mass are often used as indices of condition, which is directly related to the survival pattern of amphibians (Sinsch & Schäfer, 2016). Therefore, the quality and amount of nutrition can influence the subsequent longevity and fecundity of adults, and additional resources in the form of insects attracted by fruits may play a major role. To the best of our knowledge, little information is available on the potential role of fruit trees in facilitating food acquisition by juvenile anurans.

The main driving force of global amphibian extinction is human activity ranging from direct catching for consumption, road mortality, and destruction of habitats to indirect effects such as the spread of diseases or the growth of the ozone hole (Stuart et al., 2005; Elżanowski et al., 2009; Brühl et al., 2013; Hocking & Babbitt, 2014; Saenz, Hall & Kwiatkowski, 2014; Ficetola et al., 2015). Also, in Europe, the plight of many common species is becoming alarming due to growing human impact, despite extensive knowledge on the subject and the introduction of restrictive laws protecting amphibians (Denoël, 2012; Denoël et al., 2013; Beebee, 2014; Petrovan & Schmidt, 2016). One of the most common amphibian species in Europe, occurring along a gradient from heavily urbanised to rural, is the green toad *Bufotes viridis* (Kuzmin, 2001). This species is undergoing a decline mainly on the fringes of its range in Europe (it is listed as least concern, according to IUCN, 2018) despite its widely-known high level of adaptation to living in proximity to humans (Kuzmin, 2001; Aghasyan et al., 2015).

Considering the above information, we investigated, by conducting an enclosure experiment, whether the presence of windfalls of fleshy fruits (plums, *Prunus cerasifera*) which offer a higher level of availability to insects might have a positive effect on the post-metamorphic growth and feeding behaviour of the green toad. We tested the two following hypotheses: (1) green toad juveniles display greater growth in treatments with rotten plums *P. cerasifera* due to the presence of more varied food such as wild invertebrates; (2) green toad juveniles will be more active in the presence of fleshy fruits by increasing foraging movements as a response to prey attracted by plums.

## Materials and Methods

### Study species

The green toad *Bufotes viridis* (Laurenti, 1768) (synonym *Bufo viridis*; Bufonidae) is a widely distributed species inhabiting most of Europe, as well as central Asia as far as western Kazakhstan (Kuzmin, 2001; Aghasyan et al., 2015). In some areas, the green toad is considered a synanthropic species (see Ogielska & Kierzkowski, 2010) which prefers habitats created by humans (Mollov, 2011), rural sites, and urban habitats (Ensabella et al., 2003; Kovács & Sas, 2010; Zawadzki, Flesch & Kaczmarski, 2017). *Bufotes viridis* is known to be attracted by artificial lights and consumes insects near street lamps (Covaciu-Marcov et al., 2010). Generally, this species feeds on the most abundant potential prey in the feeding territory (and thus is considered an opportunistic predator); however, its main prey are terrestrial invertebrates, in particular ants (Baruš & Oliva, 1992; Covaciu-Marcov et al., 2010). This is why we chose it as a model species for our research.

*Prunus cerasifera* is commonly known as the cherry plum or the myrobalan plum. Native to Southeast Europe and Western Asia, it was introduced into the rest of Europe and nowadays is classified as a neophyte. Wild types include large shrubs or small trees between 8 and 12 m in height. It is one of the first European trees to flower in spring, often starting to bloom before the leaves have opened. The cherry plum (yellow or red edible drupe two to three cm in diameter) is a popular ornamental tree for garden and landscaping use; numerous cultivars have been developed (Zohary & Hopf, 2000). Spontaneous spreading trees often occur in ruderal post-industrial areas, near railroads, on the borders of fields, and as hedges or on forest edges (Colic et al., 2003; Nikolic & Rakonjac, 2007).

All of the green toad individuals used in this study derived from one population in the centre of Poznań, Wielkopolska region, Poland, 75 m a.s.l. (52°24′4″N, 16°54′54″E) (Zawadzki, Flesch & Kaczmarski, 2017). The climate is within the transition zone between humid continental and oceanic, with average summer temperatures of 18.1 °C in July, winter temperatures of −1.6 °C in January, and average annual precipitation of 517 mm (WOS 2010, after Majkowska et al., 2017). In these conditions, the annual breeding season of *B. viridis* runs from April to early summer (Kaczmarski, Szala & Kloskowski, in press). Freshly metamorphosed individuals begin to leave the breeding pond in high densities, depending on the time the eggs are deposited, from mid-July until the end of August. At the same time, plums ripen and start to fall, from early July to mid-September.

### Experimental design and procedures

Two treatments, replicated twice were used in the study. The first treatment was with fruit absent (control) in turn, the second type was with fruit present (fruit treatment). Freshly metamorphosed of green toads (120 individuals) were collected from the pond located in Poznań the same day, on 30 July 2017, at the peak of dispersion. This ensured that all individuals had emerged from the pond at the same time, signifying that they were approximately the same age. Next, toads were randomly assigned to one of four experimental groups, with 30 fresh metamorphs per group. Each group was placed in a separate enclosure with the following dimensions: 1 × 1 × 1 m. According to Kuzmin (2001), the maximum number of fresh metamorphs per one m^2^ should not exceed several dozen individuals; thus, the density during the experiment was acceptable. Each enclosure contained two numbered hiding places (built from plastic stands) and one bowl with water. The enclosures were composed of wooden frame and covered by agrotextiles in turn, the ground was filled with moist soil which created conditions appropriate for amphibians. All enclosures were covered by lids composed of the wooden frame and net with four cm mesh size. The lids protected amphibians from predators such as birds and feral cats but allowed invertebrates (mostly flying insects) to get in and out of the enclosures.

**Figure 1:**
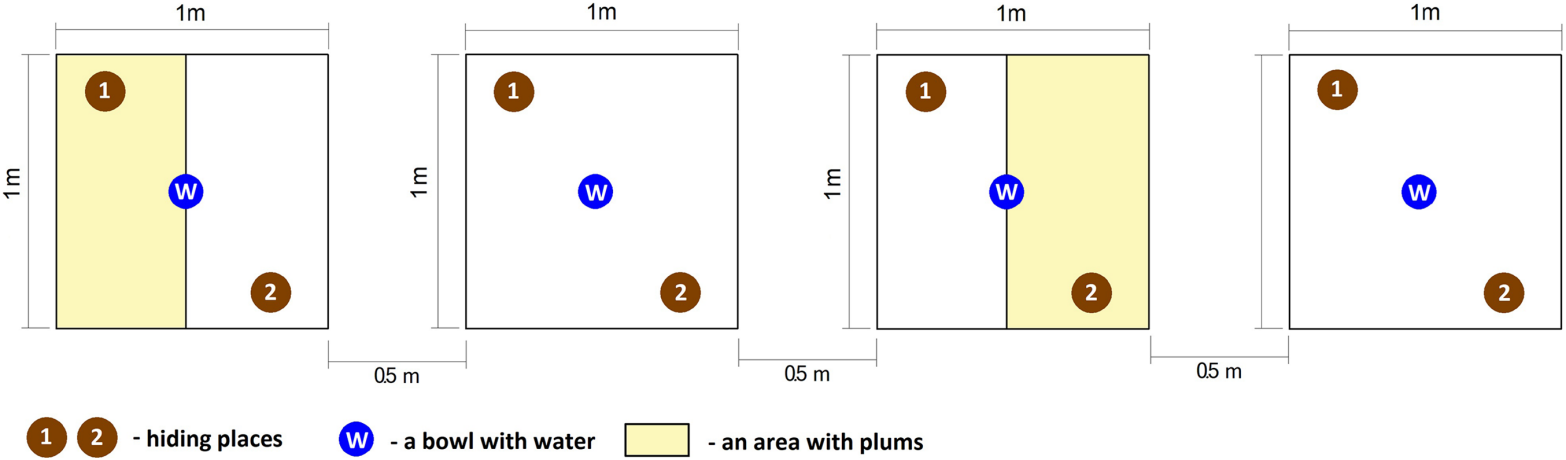
Graphical representation of experimental enclosures.

Additionally, two enclosures included plums, which were strewn about in half of the experimental area. The number of fleshy fruits in the enclosures was estimated based on the mean number of plums per one m^2^ calculated from 20 areas located in Poznań. The mean number of fruits per one m^2^ was then divided in half, since plums were strewn only on one side of the enclosure and covered 0.5 m^2^. This approach ensured us that the number of fruits reflected reality. All enclosures were placed alternately along a line in a partly shaded place so that each pair of enclosures with plums was separated by an experimental area without fleshy fruits. The distance between each pair of enclosures was 0.5 m (Fig. 1).

Throughout the experiment, body mass was recorded to the nearest 0.01 g with a portable electronic balance; snout-vent length (SVL) was measured to the nearest 0.01 mm with a digital calliper. Measurements were carried out at noon, every other day, for 30 days, and were conducted on all individuals from all groups. Both parameters were measured and recorded by the same observer during the whole experiment. Additionally, other parameters, such as temperature, humidity, numbers of active and hidden toads, numbers of missing toads, and presence of invertebrates, were noted every other day for all enclosures separately. Since the green toads were kept in enclosures, a feeding procedure was established to exclude the death from starvation of the individuals from the control group during the duration of the experiment. All groups had access to an equal amount of food. The diet comprised invertebrates such as crickets, *Callosobruchus* spp. and *Shelfordella lateralis* (with vitamin supplements). Toads were fed ad libitum. This kind of food is often used in terraria to feed frogs and toads or other insectivores (Finke & Winn, 2004; Mc Williams, 2008). The water was changed at feeding times, after all individuals were measured.

Green toad is under strict species protection in Poland; thus, the permission of the Regional Director of Environmental Protection has been obtained (No. WPB—II.6401.346.2017.AC). Therefore, we followed all applicable institutional and national guidelines for the care and use of animals. Moreover, the observer (MK) responsible for the feeding and measurement of green toads was trained by the Polish Laboratory Animals Science Association. All animals were released at the site of capture in accordance with the permission granted us.

### Statistical analysis

In order to estimate measurement error, the body mass and SVL of 30 randomly selected green toads were measured twice by one observer during the 21st day of the experiment (MK). Then, the relative technical error of measurement (relative TEM) for MK was calculated. Relative TEM expresses the quality of measurement in percentages; the lower the index of relative TEM, the greater the accuracy of the measured parameters (Perini et al., 2005). Repeated measurements were performed the same day with a 5-h break between the first and second rounds. Both measurements were performed the same day in order to avoid the influence of the toads’ development.

To check whether living conditions were the same in all experimental cages, temperature and humidity were tested for normality and homogeneity of variances using the Shapiro–Wilk and Fligner–Killeen tests, respectively. Next, differences in temperature and humidity among four cages were analysed using a paired Student’s *t*-test for which the significant level was established at *p* < 0.004 after the Bonferroni correction in order to counteract the problem of multiple comparisons.

The body mass index (BMI) was calculated according to the following equation: body mass (g)/SVL (mm)^2^ (Labocha, Schutz & Hayes, 2014). Body mass, SVL, and BMI were tested for normality (Shapiro–Wilk test) and homogeneity of variance (Levene’s test) for each group separately. In the case of *B. viridis*, the parameters could not be assigned to particular individuals due to changes in pattern and colouration during post-metamorphic growth (similar to the natterjack toad *Epidalea calamita*, according to (Meyer & Grosse, 1997)). Therefore, correlation between three parameters (SVL, body mass, and BMI) was carried out using the Pearson correlation coefficient on the mean values for all groups in aggregate.

An unpaired Student’s *t*-test for BMI was conducted to determine whether individual green toads had been randomly divided into control and experimental groups. In order to test the first hypothesis (i.e. juvenile toads display greater growth in the presence of rotten plums), general linear mixed models were used. The presence of fleshy fruits was the criterion for consideration as a treatment group. Individual measurements and day of the experiment were the random variables. Moreover, time (days of the experiment) was crossed with the presence of fleshy fruits and designated a fixed effect (Bates, 2010). In order to test the second hypothesis (i.e. juvenile toads will be more active in the presence of fleshy fruits), analysis of variance (ANOVA) was carried out. The number of active toads (individuals who were visible to the observer and did not hide in the hiding places) for every day of the experiment and the experimental areas (two enclosures with fruits and two enclosures without fruits) were considered dependent and independent variables, respectively. All statistical tests were significant at the level *p* < 0.05 and were carried out using R software (version 3.4.2, R Development Core Team, 2008).

## Results

**Figure 2:**
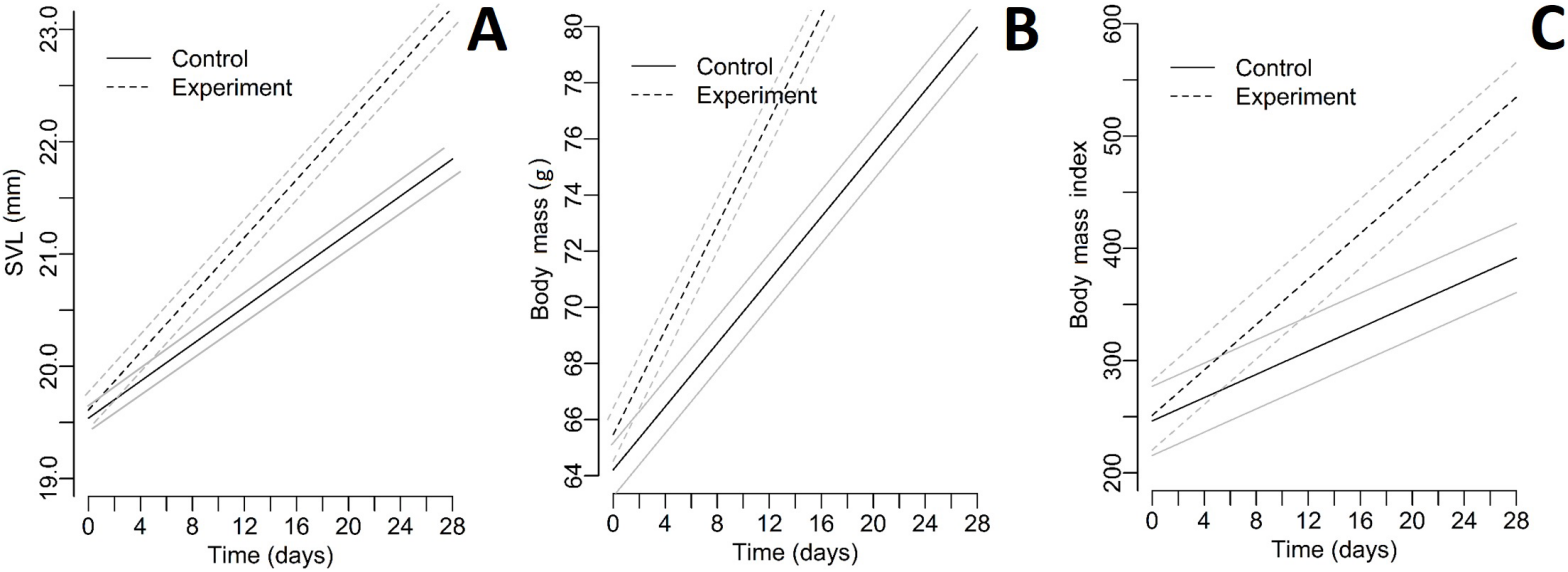
Plots representing the measurements and BMI index of toads during the experiment. (A) SVL, (B) body mass, (C) body mass index. Experiment—groups with fleshy fruits (plums); Control—groups without fleshy fruits (plums); grey line—95% confidence interval; black line—fitted line.

The relative TEM index was acceptable for both parameters (body mass = 1.43%; SVL = 2.18%). Differences in living conditions (temperature and humidity) between enclosures were not significant (all *p* > 0.004; paired Student’s *t*-test after the Bonferroni correction). Collecting all green toad juveniles was not possible twice during the experiment. Descriptive statistics of SVL, body mass, and BMI for each group during the whole experiment are included as a supplement (Tables S1–S3). All Pearson correlation coefficients were significant (Table 1). The differences in SVL, body mass, and BMI between control and experimental groups were not significant for the first day of the experiment (Table 2). The quadratic effect of time was insignificant for all dependent variables (for all *p* > 0.05); therefore, we dropped these effects from the final models. For all tested parameters (SVL, body mass, and BMI), fixed effects (time and the presence of fleshy fruits) were significant (Table 3). A graphical presentation of changing SVL, body mass, and BMI over time is shown in Fig. 2. The number of active toads did not differ significantly different between enclosures (ANOVA: *F* = 0.076, *p* = 0.972). Invertebrates were first observed in the enclosures containing fruits on Day 4 and occurrence continued through Day 28. In contrast, invertebrates were not observed in control enclosures until Day 10, and on many later occasions afterwards no invertebrates were seen. Table S4 contains the list of taxa noted during the experiment.

**Table 1:** Pearson correlation coefficients of SVL, body mass, and BMI.

	*N*	SVL	Body mass
Body mass	60	0.883*	
BMI	60	0.959*	0.975*

**Notes:**

SVL, snout-vent length, BMI, body mass index.

* Indicates significant Pearson correlation coefficient at *p* < 0.05.

**Table 2:** Student’s *t*-test between the control and experimental groups for the first day of the experiment.

	*N*	Mean (exp)	Mean (cont)	d*f*	*t*	*p*
SVL	120	18.71	18.83	118	−0.5787	0.5639
Body mass	120	0.64	0.63	114.7	0.3219	0.7481
BMI	120	225.55	226.84	117.4	−0.13122	0.8958

**Notes:**

SVL, snout-vent length; BMI, body mass index; Mean (exp), a mean value for the experimental group; Mean (cont), a mean value for the control group.

**Table 3:** Results of GLMM for SVL, body mass, and BMI of green toads.

	Estimate	Standard error	d*f*	*Z*-value	*p*
*SVL*					
Intercept	19.537	0.086			
Time	0.082	0.005	16.465	15.879	<0.001*
Exp	0.072	0.121	4.866	0.598	0.577
Time*Exp (fe)	0.046	0.007	16.555	6.223	<0.001*
Individual (*r*)	<0.001	0.010			
Time (*r*)	<0.001	<0.001			
*Body mass*					
Intercept	246.245	7.315			
Time	5.184	0.528	4.768	9.829	<0.001*
Exp	4.938	10.338	12.005	0.478	0.641
Time*Exp (fe)	3.617	0.747	4.782	4.845	0.005*
Individual (*r*)	15.690	3.961			
Time (*r*)	0.220	0.469			
*BMI*					
Intercept	64.209	1.015			
Time	0.563	0.073	4.419	7.746	0.001*
Exp	1.637	1.435	8.347	1.141	0.286
Time*Exp (fe)	0.370	0.103	4.430	3.597	0.019*
Individual (*r*)	0.483	0.695			
Time (*r*)	0.005	0.069			

**Notes:**

SVL, snout-vent length; time, next days of the experiment; Exp, type of treatment (control or experimental group of green toad juveniles); BMI, body mass index; fe, a variable as a fixed effect; *r*, variables as a random effect.

* Indicates significant variable at *p* < 0.05.

## Discussion

All of the analysed parameters (SVL, body mass, and BMI index) were greater in individuals from the two experimental groups (with fruits) than from the control groups (without fruits). Thus, the results confirmed our first hypothesis: that post-metamorphs of the green toad kept with fallen fruits (*P. cerasifera*) were characterised by faster growth than those kept without fruits. We suggest that these differences may be due to the presence of more varied food. Invertebrates are attracted generally by fallen fruits not only plums. All toads had unrestricted access to wild invertebrates, but we observed much more frequent presence of wild invertebrates in the experimental enclosures with fruits (Table S4 in the Supplemental Files). The persistence of ripe fleshy fruits on the ground is strictly dependent on the presence of frugivores and the kind/species of fruit (Tang, Corlett & Hyde, 2005). Fallen fruits start to decompose and to become attractive to many taxa of insects, such as Lepidoptera, Hemiptera, Coleoptera, Hymenoptera, or Diptera (Sallabanks & Courtney, 1992; Rodríguez, Alquézar & Peña, 2013). Fruits, when they fall from trees, are usually superabundant and may limit the intensity of competition between juvenile toads. Similarly, in experimental conditions inside enclosures insects associated with these fruits occur regularly, especially fruit flies *Drosophila* (see Table S4 in the Supplemental Files). Adult *Drosophila* are very numerous and known to be attracted by fruits (Sallabanks & Courtney, 1992). In particular, it is worth noting that fruit flies are often the primary ingredient in the diets of young amphibians bred in captivity (Dugas, Yeager & Richards-Zawacki, 2013). These invertebrates may also supplement amphibian diets which stimulate growth, especially where a significant decline in the number of insects is observed (which is disturbing, considering the important role of insectivores in the global circulation of matter) (Hallmann et al., 2017). Moreover, the great numbers of *P. cerasifera* in the environment and the lack of human interest in their fruits as a resource causes considerable periodic accumulations of fruits on the ground (between July and mid-September). Exactly at the same time, freshly metamorphosed individuals begin to leave breeding ponds in great numbers.

In our study conditions, differences in the numbers of active juvenile green toads between the experimental and control groups were not significant. Therefore, the second hypothesis, that *B. viridis* exhibits more active feeding behaviour in the presence of fleshy fruits than in their absence, could not be confirmed. It is difficult to refer to data on diet of juvenile toads in natural conditions because this type of information is very scarce and studies have usually been carried out on adults (Baruš & Oliva, 1992; Covaciu-Marcov et al., 2005, 2010). Little is known about the diet of juvenile green toads, but it is certainly similar to that preferred by adults, differing mostly in size, which is connected with a change in the foraging strategy during growth (Kuzmin, 2001). Adult green toads forage and feed intensively in the summer, unaffected by drought or high temperatures. *Bufotes viridis* consumes almost exclusively terrestrial prey (including flying insects), but also accidentally eats aquatic prey animals, meaning that the green toad can be considered a strong generalist (Covaciu-Marcov et al., 2005). Therefore, the insects attracted by fallen fruits can be treated as normal source of food for toads without affecting feeding behaviour. Windfall flashy fruit (in a broader sense, *Malus, Prunus, Pyrus, Cerasus*, and others) may be important in the lives of amphibians within a limited but crucial time period in temperate climate zones (similarly as in the case of mammals; see: Kurek, 2015).

This study is characterised by certain limitations. The first is that parameters were not assigned to particular individuals. This approach was carried out due to changes in pattern and colouration during the post-metamorphic growth of *B. viridis.* Similar changes have been observed in other toads, for example, *Epidalea calamita* (Meyer & Grosse, 1997). Assigning each parameter to individual toads might yield more information about variations in development dynamics. Therefore, in future, similar studies should also focus on analysis on an individual level in species where the assignment of parameters to particular specimens is possible. The second limitation is that no controls were carried out during the night. Fallen fruits can attract, for example, nocturnal Lepidoptera such as Catocalinae. The green toad is a nocturnal species which can be attracted by artificial lights and which consumes insects near street lamps (Covaciu-Marcov et al., 2010). Thus, night control might have yielded information about feeding behaviour and possible prey. On the other hand, additional checks at night might have been stressful and might have negatively influenced the toads’ behaviour and body condition. Moreover, the lack of night controls did not influence the obtained results, which showed faster growth in individuals from the experimental group (with plums).

Numerous studies are being undertaken to explain how urbanisation affects species (Johnson & Munshi-South, 2017). However, while the need to protect breeding ponds for the preservation of amphibian populations is well documented, it is necessary to conduct research verifying the significance of individual components of the environment, for example, constructions of embankments under tracks, in the life history of particular amphibians (Kaczmarski & Kaczmarek, 2016). Similar studies have been carried out on birds (Morelli et al., 2014). Intensively urbanised space and intensive-use agricultural and forest areas have become the dominant forms of land use. This leads to the fragmentation of natural environmental patches (Curado, Hartel & Arntzen, 2011). Introducing practical knowledge, such as a system of ecological buffers around breeding sites or key habitats, may be an effective solution (Semlitsch & Bodie, 2003; Scheele et al., 2014).

Fruit trees are an important component of suburban or rural flora, (1) from both an aesthetic (as an ornamental tree) and practical points of view; (2) because they constitute food resources; and in a broader sense, (3) because they provide ecosystem services (Giergiczny & Kronenberg, 2014; Lafontaine-Messier, Gélinas & Olivier, 2016; Larondelle & Strohbach, 2016). Some fruit tree species can attract pests, for example, rats (*Rattus*), flies (*Musca*), and wasps (*Vespa* and *Vespula*); on the other hand, the appearance of masses of insects attracted by fermenting fruit also affects other predators on invertebrates, such as mammals, birds, and lizards (Sallabanks & Courtney, 1992; Rodríguez, Alquézar & Peña, 2013). Our study showed that green toad juveniles can also benefit from the presence of invertebrates attracted by fermenting fruits. However, fallen fruits and the species associated with them are treated by city administrators, as well as residents themselves, as a problem (e.g. shade, odour, the need to remove leaves) (Kronenberg, 2012) and even as a biological threat (e.g. pathogen vectors, painful stings). In our opinion, fruit trees, along with the insects they periodically attract, should begin to serve as a kind of keystone for urbanised and ruderal areas. This aspect is important, especially since trees are currently being removed from the urban environment as a result of political and social pressure to maintain cleanliness (Kronenberg, 2012; Andrew & Slater, 2014; Conway, 2016).

As far as we know, this study is the first to focus on the indirect role of fruit trees as a source of food for amphibians at a critical moment of their development, that is, after metamorphosis and during the period of intensive growth preceding the first hibernation. This issue suggests several additional possibilities concerning the influence of fallen fruits on growth dynamics on an individual level and the distribution of amphibians in the environment. However, fruit lying on the ground can constitute an ecological trap: amphibians attracted by insects can be eaten by predators, such as wild boars (*Sus scrofa*) feeding on fallen fruits. Similarly, predation by ground beetles (Carabid) may be a threat to group of amphibians feeding on fruits (Wizen & Gasith, 2011; Schaefer et al., 2018). Particularly important is the question of whether the distribution of fruit trees causes periodic aggregations of amphibians and affects their post-breeding dispersion. If so, properly spaced fruit trees (greenery design) can contribute to reducing the road mortality of amphibians by redirecting them. This kind of hypothetical effect may have enormous potential for the conservation of amphibians. Moreover, in a broader sense, indicating a ‘new’ function for trees (*sensu stricto*: ecosystem services) may have a positive impact on local biodiversity in urban and rural areas.

## Conclusion

Toads from experimental enclosures with *P. cerasifera* were characterised by faster growth than individuals from the experimental area without fleshy fruits. The falling fleshy fruits attract many types of invertebrates that can be eaten by toads; therefore, the presence of fruit trees close to a breeding site might influence positively the post-metamorphic growth or dispersion of amphibians. However, it is necessary to test these assumptions in natural conditions and in other amphibian species. No differences in active feeding behaviour were noted between experimental groups with and without *P. cerasifera* fruits, which means that fallen fruits can be treated as an attractor of food (e.g. insects) without affecting feeding behaviour. To our knowledge, no previous study has investigated the potential influence of the presence of fruit trees on the growth and behaviour of anurans.

## Supplemental Information

10.7717/peerj.6337/supp-1 Supplemental Information 1 Descriptive statistics for SVL [mm] measurement in all groups of green toads.

10.7717/peerj.6337/supp-2 Supplemental Information 2 Descriptive statistics for BMI index in all groups of green toads.

10.7717/peerj.6337/supp-3 Supplemental Information 3 Descriptive statistics for body mass weight [g] measurement in all groups of green toads.

10.7717/peerj.6337/supp-4 Supplemental Information 4 List of invertebrates noted during the experiment.

10.7717/peerj.6337/supp-5 Supplemental Information 5 Amphibian measurements and experimental design details. Column B–snout-vent length (SVL); Column C–body mass; Column D–body mass index (BMI)(body mass[g]/SVL[mm]^2^); Column D–number of enclosures (K1–K4) and treatmens (M1, M2); Column E–comments; Column F–date; Column H–experimental day.

## Additional Information and Declarations

### Field Study Permissions

The following information was supplied relating to field study approvals (i.e. approving body and any reference numbers): Experiments were approved by the Regional Director of Environmental Protection (permission no. WPB—II.6401.346.2017.AC).

### Data Availability

The following information was supplied regarding data availability: The raw measurements are provided in the raw data file provided in the Supplemental Files.
