# Supplementary material for: Urban plums and toads: do fleshy fruits affect the post-metamorphic growth of amphibians?
Source: PeerJ. 2019 Jan 30;7:e6337. doi: 10.7717/peerj.6337 (PMC6359899; doi:10.7717/peerj.6337)
Supplement: Supplemental Information 1 [file peerj-07-6337-s001.docx]

**Supplemental Table S1. Descriptive statistics for SVL [mm] measurement in all groups of green toads**

| Day | M | Min | Max | SD | M | Min | Max | SD | M | Min | Max | SD | M | Min | Max | SD |
| --- | --- | --- | --- | --- | --- | --- | --- | --- | --- | --- | --- | --- | --- | --- | --- | --- |
|  | Plums 1 | | | | Plums 2 | | | | Control 1 | | | | Control 2 | | | |
| 0 | 18.83 | 17.23 | 21.59 | 1.13 | 18.60 | 16.85 | 20.92 | 1.09 | 19.12 | 16.48 | 20.66 | 1.08 | 18.54 | 16.51 | 20.32 | 1.08 |
| 2 | 20.22 | 18.48 | 22.32 | 1.12 | 19.49 | 16.52 | 20.95 | 1.05 | 19.58 | 17.10 | 21.83 | 1.18 | 19.92 | 16.68 | 22.30 | 1.18 |
| 4 | 20.57 | 18.06 | 23.56 | 1.27 | 19.98 | 18.06 | 21.40 | 0.85 | 20.34 | 18.11 | 22.49 | 0.99 | 20.05 | 17.04 | 21.98 | 1.10 |
| 6 | 20.09 | 18.92 | 22.42 | 0.97 | 20.50 | 17.74 | 22.47 | 0.98 | 19.77 | 17.72 | 23.00 | 1.08 | 19.88 | 17.52 | 22.69 | 1.33 |
| 8 | 21.06 | 19.48 | 23.18 | 0.97 | 20.77 | 18.38 | 22.31 | 0.92 | 20.34 | 17.74 | 22.94 | 1.04 | 20.07 | 16.77 | 22.95 | 1.23 |
| 10 | 21.44 | 18.98 | 24.35 | 1.34 | 21.24 | 18.10 | 22.79 | 1.08 | 20.87 | 18.24 | 23.42 | 1.22 | 20.86 | 17.51 | 23.17 | 1.42 |
| 12 | 21.26 | 19.82 | 23.82 | 1.15 | 21.35 | 18.24 | 23.19 | 1.13 | 20.50 | 16.99 | 23.50 | 1.50 | 20.79 | 17.56 | 23.98 | 1.43 |
| 14 | 21.61 | 19.82 | 24.44 | 1.27 | 21.68 | 17.94 | 23.54 | 1.22 | 20.85 | 18.54 | 23.68 | 1.16 | 20.81 | 17.41 | 24.16 | 1.57 |
| 16 | 21.90 | 19.49 | 25.37 | 1.44 | 22.04 | 17.99 | 23.77 | 1.41 | 21.07 | 19.17 | 23.70 | 1.10 | 21.05 | 17.31 | 25.25 | 1.72 |
| 18 | 22.22 | 20.14 | 25.24 | 1.32 | 21.99 | 18.33 | 24.56 | 1.24 | 21.29 | 19.07 | 24.21 | 1.13 | 21.15 | 18.13 | 24.86 | 1.59 |
| 20 | 22.35 | 20.01 | 25.86 | 1.33 | 22.08 | 18.22 | 24.34 | 1.23 | 21.20 | 18.70 | 24.00 | 1.33 | 21.09 | 16.98 | 24.29 | 1.62 |
| 22 | 22.17 | 19.67 | 25.57 | 1.34 | 22.33 | 18.19 | 24.88 | 1.38 | 21.28 | 18.70 | 24.10 | 1.18 | 21.23 | 17.26 | 25.30 | 1.65 |
| 24 | 22.64 | 20.09 | 26.11 | 1.48 | 22.35 | 16.46 | 25.51 | 1.60 | 21.17 | 18.07 | 24.33 | 1.26 | 21.28 | 17.18 | 25.28 | 1.81 |
| 26 | 22.78 | 20.11 | 26.03 | 1.49 | 22.84 | 18.31 | 24.98 | 1.44 | 21.62 | 18.91 | 24.52 | 1.41 | 21.80 | 16.99 | 25.60 | 1.95 |
| 28 | 22.96 | 20.19 | 25.34 | 1.32 | 22.74 | 16.71 | 25.45 | 1.67 | 21.42 | 18.91 | 24.23 | 1.30 | 21.84 | 16.07 | 25.64 | 1.99 |

M – mean, SD – standard deviation, Plums 1 – first group of green toads with plums, Plums 2 – second group of green toads with plums, Control 1 – first control group of green toads, Control 2 – second control group of green toads
