# Supplementary material for: Urban plums and toads: do fleshy fruits affect the post-metamorphic growth of amphibians?
Source: PeerJ. 2019 Jan 30;7:e6337. doi: 10.7717/peerj.6337 (PMC6359899; doi:10.7717/peerj.6337)
Supplement: Supplemental Information 2 [file peerj-07-6337-s002.docx]

**Supplemental Table S2. Descriptive statistics for body mass weight [g] measurement in all groups of green toads**

| Day | M | Min | Max | SD | M | Min | Max | SD | M | Min | Max | SD | M | Min | Max | SD |
| --- | --- | --- | --- | --- | --- | --- | --- | --- | --- | --- | --- | --- | --- | --- | --- | --- |
|  | Plums 1 | | | | Plums 2 | | | | Control 1 | | | | Control 2 | | | |
| 0 | 0.65 | 0.51 | 0.89 | 0.10 | 0.62 | 0.43 | 0.72 | 0.07 | 0.66 | 0.47 | 0.84 | 0.09 | 0.60 | 0.39 | 0.79 | 0.09 |
| 2 | 0.70 | 0.55 | 0.96 | 0.10 | 0.67 | 0.43 | 0.81 | 0.08 | 0.69 | 0.52 | 1.00 | 0.11 | 0.68 | 0.50 | 0.89 | 0.10 |
| 4 | 0.72 | 0.57 | 0.99 | 0.11 | 0.68 | 0.47 | 0.84 | 0.08 | 0.69 | 0.53 | 0.89 | 0.10 | 0.68 | 0.46 | 0.89 | 0.10 |
| 6 | 0.62 | 0.47 | 0.92 | 0.10 | 0.70 | 0.50 | 0.97 | 0.10 | 0.63 | 0.43 | 0.85 | 0.10 | 0.63 | 0.43 | 0.93 | 0.13 |
| 8 | 0.78 | 0.60 | 1.09 | 0.13 | 0.77 | 0.49 | 0.95 | 0.10 | 0.69 | 0.49 | 1.04 | 0.12 | 0.70 | 0.47 | 0.96 | 0.11 |
| 10 | 0.78 | 0.57 | 1.25 | 0.14 | 0.80 | 0.47 | 0.96 | 0.11 | 0.72 | 0.49 | 0.96 | 0.11 | 0.75 | 0.48 | 1.06 | 0.14 |
| 12 | 0.80 | 0.63 | 1.19 | 0.14 | 0.76 | 0.53 | 1.03 | 0.11 | 0.69 | 0.49 | 1.01 | 0.12 | 0.70 | 0.45 | 1.05 | 0.15 |
| 14 | 0.77 | 0.60 | 1.15 | 0.14 | 0.76 | 0.50 | 1.01 | 0.11 | 0.69 | 0.50 | 0.96 | 0.11 | 0.65 | 0.43 | 0.96 | 0.14 |
| 16 | 0.77 | 0.60 | 1.14 | 0.14 | 0.80 | 0.47 | 1.04 | 0.12 | 0.67 | 0.47 | 0.92 | 0.11 | 0.69 | 0.45 | 1.07 | 0.16 |
| 18 | 0.85 | 0.56 | 1.29 | 0.17 | 0.83 | 0.46 | 1.11 | 0.14 | 0.76 | 0.48 | 1.28 | 0.15 | 0.75 | 0.43 | 1.13 | 0.16 |
| 20 | 0.90 | 0.66 | 1.37 | 0.16 | 0.94 | 0.52 | 1.28 | 0.15 | 0.83 | 0.52 | 1.32 | 0.15 | 0.89 | 0.50 | 1.28 | 0.17 |
| 22 | 0.86 | 0.59 | 1.35 | 0.18 | 0.85 | 0.44 | 1.11 | 0.14 | 0.75 | 0.52 | 1.03 | 0.12 | 0.79 | 0.38 | 1.25 | 0.19 |
| 24 | 0.86 | 0.58 | 1.39 | 0.18 | 0.85 | 0.38 | 1.18 | 0.16 | 0.73 | 0.46 | 1.06 | 0.14 | 0.75 | 0.37 | 1.15 | 0.18 |
| 26 | 0.91 | 0.57 | 1.32 | 0.16 | 0.89 | 0.48 | 1.18 | 0.15 | 0.79 | 0.55 | 1.18 | 0.14 | 0.83 | 0.37 | 1.30 | 0.20 |
| 28 | 0.90 | 0.60 | 1.30 | 0.19 | 0.90 | 0.42 | 1.18 | 0.17 | 0.75 | 0.47 | 1.05 | 0.14 | 0.80 | 0.34 | 1.28 | 0.19 |

M – mean, SD – standard deviation, Plums 1 – first group of green toads with plums, Plums 2 – second group of green toads with plums, Control 1 – first control group of green toads, Control 2 – second control group of green toads
