# Supplementary material for: Urban plums and toads: do fleshy fruits affect the post-metamorphic growth of amphibians?
Source: PeerJ. 2019 Jan 30;7:e6337. doi: 10.7717/peerj.6337 (PMC6359899; doi:10.7717/peerj.6337)
Supplement: Supplemental Information 3 [file peerj-07-6337-s003.docx]

**Supplemental Table S3. Descriptive statistics for BMI index in all groups of green toads**

| Day | M | Min | Max | SD | M | Min | Max | SD | M | Min | Max | SD | M | Min | Max | SD |
| --- | --- | --- | --- | --- | --- | --- | --- | --- | --- | --- | --- | --- | --- | --- | --- | --- |
|  | Plums 1 | | | | Plums 2 | | | | Control 1 | | | | Control 2 | | | |
| 0 | 234.01 | 156.19 | 368.24 | 59.33 | 217.08 | 127.66 | 301.98 | 42.87 | 245.09 | 148.34 | 343.06 | 54.39 | 208.59 | 112.84 | 305.25 | 52.27 |
| 2 | 289.14 | 194.19 | 455.81 | 71.59 | 256.01 | 117.35 | 333.46 | 50.72 | 267.33 | 155.63 | 476.55 | 66.91 | 274.60 | 176.24 | 442.59 | 67.52 |
| 4 | 311.15 | 195.70 | 485.83 | 78.60 | 274.17 | 158.09 | 348.05 | 48.34 | 287.65 | 176.91 | 450.16 | 65.05 | 277.39 | 133.57 | 415.48 | 68.14 |
| 6 | 254.43 | 176.76 | 407.65 | 64.85 | 299.45 | 163.65 | 489.75 | 63.47 | 248.63 | 141.34 | 449.65 | 62.58 | 255.67 | 131.99 | 469.56 | 85.50 |
| 8 | 352.45 | 240.86 | 585.67 | 89.22 | 333.43 | 165.53 | 448.17 | 64.39 | 290.89 | 154.21 | 507.49 | 78.11 | 286.91 | 132.18 | 505.63 | 73.88 |
| 10 | 368.00 | 214.08 | 726.01 | 112.50 | 364.28 | 153.98 | 477.83 | 75.34 | 320.43 | 191.91 | 512.95 | 85.71 | 334.04 | 147.17 | 558.30 | 104.44 |
| 12 | 368.58 | 247.48 | 651.60 | 104.35 | 350.14 | 176.33 | 553.91 | 79.29 | 298.39 | 144.33 | 524.64 | 93.12 | 313.89 | 148.01 | 596.76 | 113.17 |
| 14 | 365.31 | 240.00 | 686.91 | 111.54 | 363.66 | 160.92 | 559.67 | 82.47 | 304.51 | 171.87 | 538.31 | 79.94 | 289.91 | 130.34 | 560.36 | 108.27 |
| 16 | 376.53 | 229.09 | 733.75 | 117.65 | 403.00 | 232.58 | 574.34 | 89.15 | 299.98 | 180.07 | 483.72 | 79.14 | 320.51 | 134.84 | 682.19 | 132.79 |
| 18 | 428.37 | 246.02 | 821.80 | 139.33 | 414.03 | 223.09 | 669.54 | 88.08 | 352.71 | 174.56 | 750.24 | 109.53 | 347.77 | 141.34 | 698.36 | 130.11 |
| 20 | 461.92 | 276.28 | 916.17 | 143.36 | 467.86 | 172.62 | 758.32 | 115.15 | 380.83 | 181.84 | 752.11 | 116.61 | 409.97 | 144.16 | 753.34 | 141.54 |
| 22 | 431.94 | 240.51 | 882.66 | 142.93 | 432.01 | 145.59 | 687.11 | 112.74 | 346.70 | 181.84 | 598.23 | 93.54 | 369.71 | 113.20 | 800.11 | 151.31 |
| 24 | 453.69 | 234.09 | 947.61 | 157.24 | 434.50 | 102.95 | 767.90 | 124.99 | 332.62 | 150.20 | 621.55 | 101.46 | 356.65 | 109.21 | 717.03 | 149.89 |
| 26 | 485.90 | 230.51 | 891.63 | 149.19 | 475.19 | 160.92 | 736.32 | 124.01 | 379.00 | 214.55 | 709.45 | 111.23 | 413.67 | 106.80 | 851.97 | 171.58 |
| 28 | 485.96 | 258.84 | 834.75 | 155.02 | 477.19 | 117.27 | 764.29 | 138.85 | 350.72 | 168.07 | 616.45 | 101.32 | 401.11 | 87.80 | 841.48 | 164.90 |

M – mean, SD – standard deviation, Plums 1 – first group of green toads with plums, Plums 2 – second group of green toads with plums, Control 1 – first control group of green toads, Control 2 – second control group of green toads
