# Supplementary material for: Urban plums and toads: do fleshy fruits affect the post-metamorphic growth of amphibians?
Source: PeerJ. 2019 Jan 30;7:e6337. doi: 10.7717/peerj.6337 (PMC6359899; doi:10.7717/peerj.6337)
Supplement: Supplemental Information 4 [file peerj-07-6337-s004.docx]

**Supplemental Table S4. List of invertebrates noted during the experiment**

| Day | Plums 1 | Plums 2 | Control 1 | Control 2 |
| --- | --- | --- | --- | --- |
| 0 | no invertebrates | no invertebrates | no invertebrates | no invertebrates |
| 2 | no invertebrates | no invertebrates | no invertebrates | no invertebrates |
| 4 | *Apis mellifera*  *Drosophila*  Araneae  Muscidae | *Drosophila*  Araneae  Muscidae | no invertebrates | no invertebrates |
| 6 | *Drosophila* (numerous)  Muscidae | *Vespa crabro*  Coleoptera | no invertebrates | no invertebrates |
| 8 | no invertebrates | no invertebrates | no invertebrates | no invertebrates |
| 10 | *Vespula vulgaris* | *Drosophila* | Phalangiidae | *Culex pipiens* |
| 12 | *Apis mellifera*  *Vespula vulgaris* *Drosophila* (numerous) | *Vespula vulgaris*  *Drosophila*  Muscidae | Muscidae | no invertebrates |
| 14 | Pulmonata | *Vespula vulgaris*  *Drosophila*  Coleoptera | no invertebrates | *Forficula auricularia* |
| 16 | *Drosophila*  Muscidae | *Drosophila*  Muscidae  Phalangiidae | no invertebrates | *Porcellio scaber*  Pulmonata |
| 18 | *Drosophila* | *Drosophila*  Muscidae | no invertebrates | Pulmonata |
| 20 | *Apis mellifera*  *Drosophila* | *Drosophila* | no invertebrates | no invertebrates |
| 22 | *Vespula vulgaris* *Drosophila*  Formicidae  Muscidae (dead)  Coleoptera | *Drosophila*  Muscidae (dead) | *Drosophila* | no invertebrates |
| 24 | *Apis mellifera*  *Drosophila*  Formicidae | *Drosophila* (numerous)  Muscidae  Pulmonata | no invertebrates | *Drosophila* |
| 26 | *Vespula vulgaris*  *Drosophila* (numerous) Muscidae  Pulmonata | *Drosophila* (numerous) | no invertebrates | *Culex pipiens*  Pulmonata |
| 28 | *Nicrophorus vespillo Drosophila (numerous)* | *Drosophila* (numerous) | no invertebrates | no invertebrates |

Plums 1 – first group of green toads with plums, Plums 2 – second groups of green toads with plums, Control 1 – first control group of green toads, Control 2 – second control group of green toads.
